# Using surveillance data to understand cancer trends: an overview in Morocco

**DOI:** 10.1186/s13690-015-0094-8

**Published:** 2015-11-02

**Authors:** M. Obtel, B. Lyoussi, N. Tachfouti, S. Mathoulin Pelissier, C. Nejjari

**Affiliations:** Directorate of Epidemiology and Diseases Control, Ministry of Health, Rabat, Morocco; Laboratory of Physiology, Pharmacology and Environmental Health, Faculty of Sciences Dhar El Mahraz, Sidi Mohamed Ben Abdellah University, Fez, Morocco; Departement of Epidemiology, Clinical Research and Public Health, Faculty of Medicine and Pharmacy, Fez, Morocco; Axe Cancer, INSERM 897 ISPED, Bordeaux, France; Unit of Research and Clinical Epidemiology, Bergonié Institute, Bordeaux, France; University of Bordeaux, Bordeaux, France; Public Health School, Ministry of Health, Rabat, Morocco

**Keywords:** Cancer registry, Incidence, Mortality, Morbidity, Morocco, Registre des cancers, Incidence, Mortalité, Morbidité, Maroc

## Abstract

**Background:**

The aim was to use the existing surveillance data sources of cancer in Morocco that could be used to better describe cancer mortality and incidence trends in Morocco.

**Methods:**

National incidence data were derived from population-based cancer registries. Mortality data were collected from the international GLOBOCAN database.

**Results:**

An overview of the main results was presented. In general, the most commonly diagnosed cancers in men are lung and prostates whereas in women, breast and cervical cancers are the pre-dominant cancers. Fifty nine percent and of breast and 65.7 % of cervical cancers in women are diagnosed at stages II and III. Cancer remains the second highest cause of mortality in Morocco.

**Conclusion:**

The data provides a description of the cancer incidence and trends in the Moroccan population. The Moroccan national cancer program should aim for more coherent, consistent and comparable incidence data between different cancer registries in the country, and develop uniform datasets with respect to quality.

## Résumé

**Introduction:**

L'objectif était d'utiliser les sources de données existantes de surveillance du cancer qui pourraient être utiles pour décrire les tendances d’incidence et de mortalité du cancer au Maroc.

**Méthodes:**

Les données de morbidité et mortalité nationales et internationales disponibles ont été explorées. Les registres populationnels de cancer couvrent les données d'incidence régionale. Les données de mortalité sont disponibles par les données internationales de Globocan.

**Résultats:**

Un aperçu des principaux résultats a été présenté. Globalement, les cancers les plus fréquents sont le poumon et la prostate chez les hommes; le sein et le col utérin chez les femmes; ces deux cancers représentant 56,3 % de cancers féminins. Les cancers de sein et du col sont diagnostiqués dans 59 % et 65,7 % aux stades II et III. Le cancer est la deuxième cause de mortalité au Maroc.

**Conclusion:**

Les données fournissent une description de l'incidence des cancers et leurs tendances dans la population marocaine. Le programme national du cancer marocaine devrait viser pour les données d'incidence cohérents et comparables entre les différents registres du cancer dans le pays, et de développer des ensembles de données uniformes par rapport à la qualité.

## Background

Cancer is increasingly growing as a major health problem in both developed and developing countries amongst the chronic diseases [1]. Cancer imposes a heavy economic, health, and social burden. It is a global pandemic affecting both developed and developing regions, but the cancer toll is rapidly increasing in low and middle-income countries, where resources for prevention, diagnosis and treatment are limited or nonexistent [2]. Worldwide, cancer incidence could potentially increase to as many as 17 million new cases per year by 2020. Of these, it is estimated that as many as 1.5 million cases will occur alone in Africa [3].

The professional oncology community is being increasingly called upon to define pragmatic and realistic approaches in order to provide decision makers with the evidence that cancer is increasingly growing a major public health problem and provide information that can be used to monitor its public health threats to general population [4].

Hospital registries and surveillance systems that collect information on new cases, trends, diagnosis, treatment and mortality due to cancer in a systematic and reliable manner are key to build cancer prevention and control programme.

Data from cancer registries provide a description of the disease burden by population and over time, but geographic heterogeneities also need to be understood to develop and improve rational cancer control policies. The Middle East Africa (MEA) countries have their own patterns [5], and Morocco is among the countries that have a particular demographic and epidemiological situation. The estimated population of Morocco is around 32.6 million people, with a life expectancy at birth of 74.8 years [6]. 6.8 % of subjects are estimated to be over 65 and 29 % of the population is under 15 years old. The young population in Morocco is associated with a high fertility rate of approximately 2.59 births per woman during the period 2006–2011 [7]. In March 2010, Morocco officially launched the National Plan of Cancer Control, aiming at reducing morbidity and mortality rates and improving survival and quality of life of cancer patients by promoting prevention, early detection and effective screening practice; by improving diagnosis, treatment, and palliative care services; and by supporting the development of facilities and research for cancer throughout the country [8].

The aim of the current study was to use the existing surveillance data sources of cancer to describe cancer mortality and incidence trends in Morocco and provide an overview of cancer profile in Morocco.

## Methods

Morbidity and mortality statistics on cancer were obtained from national and internationaldata sources: whereas national data on cancer were mainly provided by the Moroccan cancer registries, international estimates are mainly based on data available from the IARC.

### National morbidity data sources

The main sources of cancer morbidity data are population-based cancer registries. In Morocco, two population-based registries are the principal sources of reliable cancer morbidity data. The first one is region-based and concerns the administrative region of Casablanca (Region du Grand Casablanca). This registry covers around 12 % of the Moroccan population and is usually tied to state health departments of the region. The data are collected retrospectively on cancer patient from private and public care structures in the Casablanca region, such as hospital departments, private hospitals and pathological laboratories [9]. This data collection is made by medical doctors who are well trained on cancer registration and who are also involved in the management of database after the collection. The first report of this registry published cancer data from 2004 [10] and a more recent publication has covered the period from 2005–2007 [11].

The second one, the population-based cancer registry of Rabat, covering around 2.1 % of the Moroccan populationand this registry reports all new cancer casesfrom 2005 onwards among residents in Rabat [12]. This registry usesan active method of data collection consistingofgathering data relative to any new diagnosed cancer including visits by registry staff to all data sources, essentially hospitals, pathological laboratories, and private clinics in Rabat [12]. Themost recent report of this registry published data for the period from 2006–2008 [13].

Data from these two population-based registries allows to estimatethe global and national incidence cancer rate and the incidence rates by localization. To compare data at national or international level, these registries estimate a standardized incidence by using the Moroccan population and the world populationas references [9, 12]. Data collected by these cancer registries usually include date of diagnosis, confirmation of diagnosis, histological type and differentiation, extension of the tumor, classification, stage and treatment, as well as socio-demographic information. Another registry of the oncology hospital hasbeen developed in the region of Marrakech, but the results have not been published yet. In theFez region, a cancer registry of Hassan II Hospitalis currently being developed to collect data and to evaluate patient care in this north centre region of Morocco. They can also be used in studies that compare patterns of care among providers or geographic regions [14].

### National mortality data sources

The main sources are data published annually by the Ministry of Health [7]. The information is obtained from death certificates completed by medical health workers of the Municipal Hygiene Office throughout the country since 1950. Currently, no data related to the cause of death according to International Classification of Diseases (ICD)10 is available in the conventional system of surveillance.

### International morbidity and mortality data sources

We used the CancerMondialwebsite to get access to various databases containing information on the occurrence of cancer worldwide held and managed by the Section of Cancer Information of IARC. Two of the principal cancer databases available on this website are GLOBOCAN, which provides the most recent estimates for 2012’s cancer incidence, mortality rate,and prevalence for 28types of cancer in 184 countries [2], and Cancer Incidence in Five Continents (CI5), which provides access to detailed information on the incidence of cancer recorded by cancer registries (regional or national) worldwide [15]. For Morocco, as for other countries, data on cancer incidence and mortality are available on the GLOBOCAN. These data are collected by IARC [2]. The datais based on provisional estimates of cancer andspecific mortality in every country, and it allows making predictions of mortality and morbidity until the year 2035. The cancer data concerning incidence, morbidity and population survival analysis based onthe Casablanca cancer registry are not yet available on the CI5 database.

## Results

### Main morbidity and mortality data

Between 2005 and 2007, the Casablanca cancer population-based registry recorded 11,923 cases (all cancer localizations), of which 5,551 cases (46.6 %) were recorded in males [9]. The incidence rates (CR: Crude rate, ASR (M): Age Standardized Rate in Morocco, ASR (W): Age Standardized Rate in the World) of global cancers are displayed in Table 1. All rates tended to increase in both men and women;the ASR (W) passed from 100.3/100000 in 2004 to 138.5/100000 in 2008 (Table 1).

**Table 1:** Cancer incidence, per 100 000 inhabitants, in Morocco according to gender and years (2004–2008: data from Casablanca and Rabat cancer registries) [9, 12]

		2004		2005		2006		2007		2008	
Registry		Men	Women	Men	Women	Men	Women	Men	Women	Men	Women
Casablanca	Case	1503	1833	1680	1994	1788	2091	2083	2287	-	-
	CR	84.0	100.0	93.1	107.4	97.8	111.0	112.4	119.8	-	-
	ASR (M)	-	-	88.8	95.1	94.0	99.7	106.6	107.5	-	-
	ASR (W)	100.3	104.2	112.2	110.6	117.1	115.1	132.8	121.9	-	-
Rabat	Cases	-	-	384	379	401	394	410	434	430	404
	CR	-	-	125.5	115.9	130.5	119.5	132.6	130.4	138.3	120.5
	ASR (M)	-	-	104.8	96.0	107.6	97.2	110.2	106.3	114.6	97.7
	ASR (W)	-	-	132.9	112.2	134.9	112.1	137.0	120.7	138.5	110.9

*CR* Crude rate, *ASR (M)* Age Standardized Rate (incidence standardized on Morocco population), *ASR (W)* Age Standardized Rate (incidence standardized on World population)

**Table 2:** Cancer incidence, per 100 000 inhabitants,of the most commonly found cancers in Moroccan population according to gender and years, (2004–2007: data from Casablanca cancer registry) [9]

	Women					Men			
	2004	2005	2006	2007		2004	2005	2006	2007
Breast					Lung				
Case	662	666	690	763	Case	357	373	380	389
ASR (M)	35.0	35.1	35.4	38.6	ASR (M)	25.5	26.0	25.9	25.9
Cervix					Prostate				
Case	235	272	276	268	Case	124	169	172	207
AS (M)	13.5	15.5	14.9	14.6	ASR (M)	9.6	12.7	12.3	15.6
Thyroid					Bladder				
Case	97	128	125	148	Case	84	107	113	148
ASR (M)	4.8	6.5	6.5	7.1	ASR (M)	5.8	8.0	7.9	10.2
Colorectal					Colorectal				
Case	93	96	100	110	Case	102	106	137	134
ASR (M)	5.8	5.6	5.9	6.0	AS (M)	6.5	6.9	9.2	8.4

*ASR (M)* Age Standardized Rate (Standardized incidence on Morocco population)

**Fig. 1:**
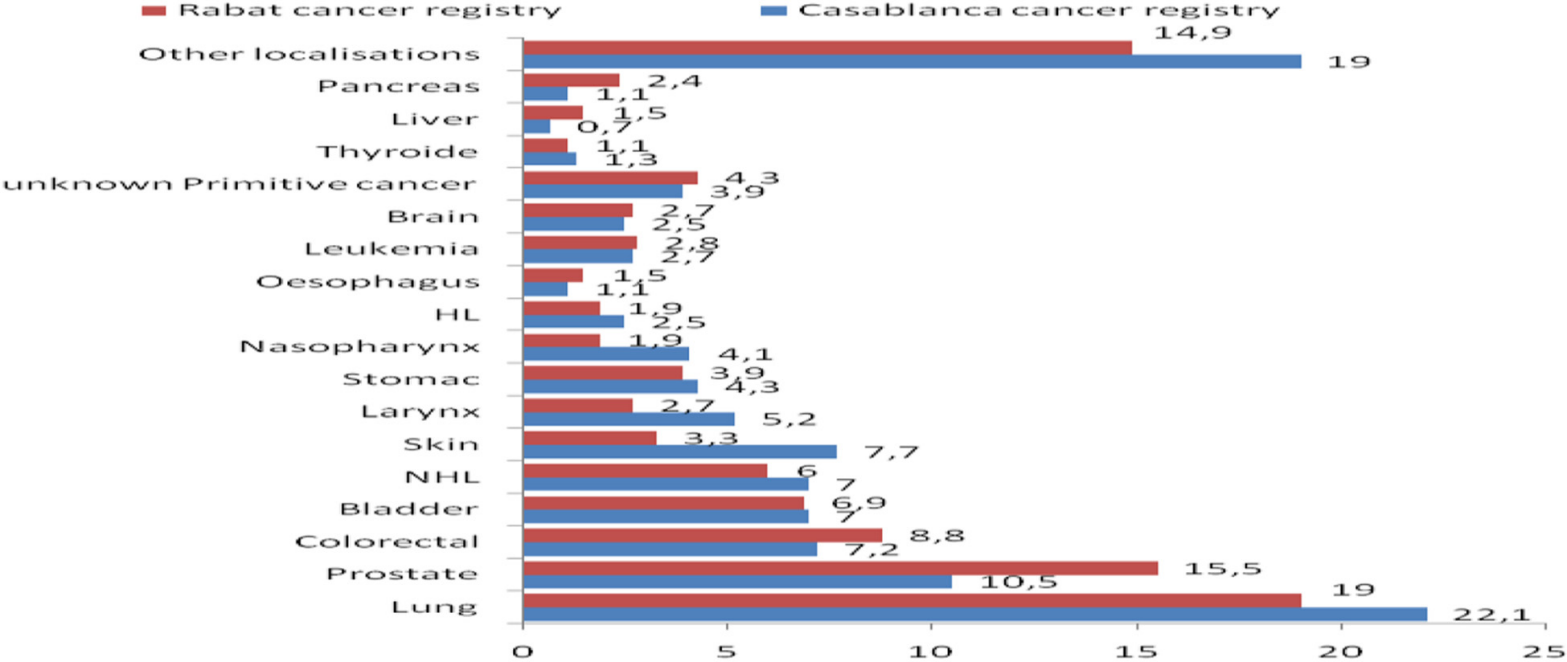
Proportion of cancer cases recorded in men, taken from Casablanca (2005-2007) and Rabat (2006-2008) population- based cancer registries [9, 12]

**Fig. 2:**
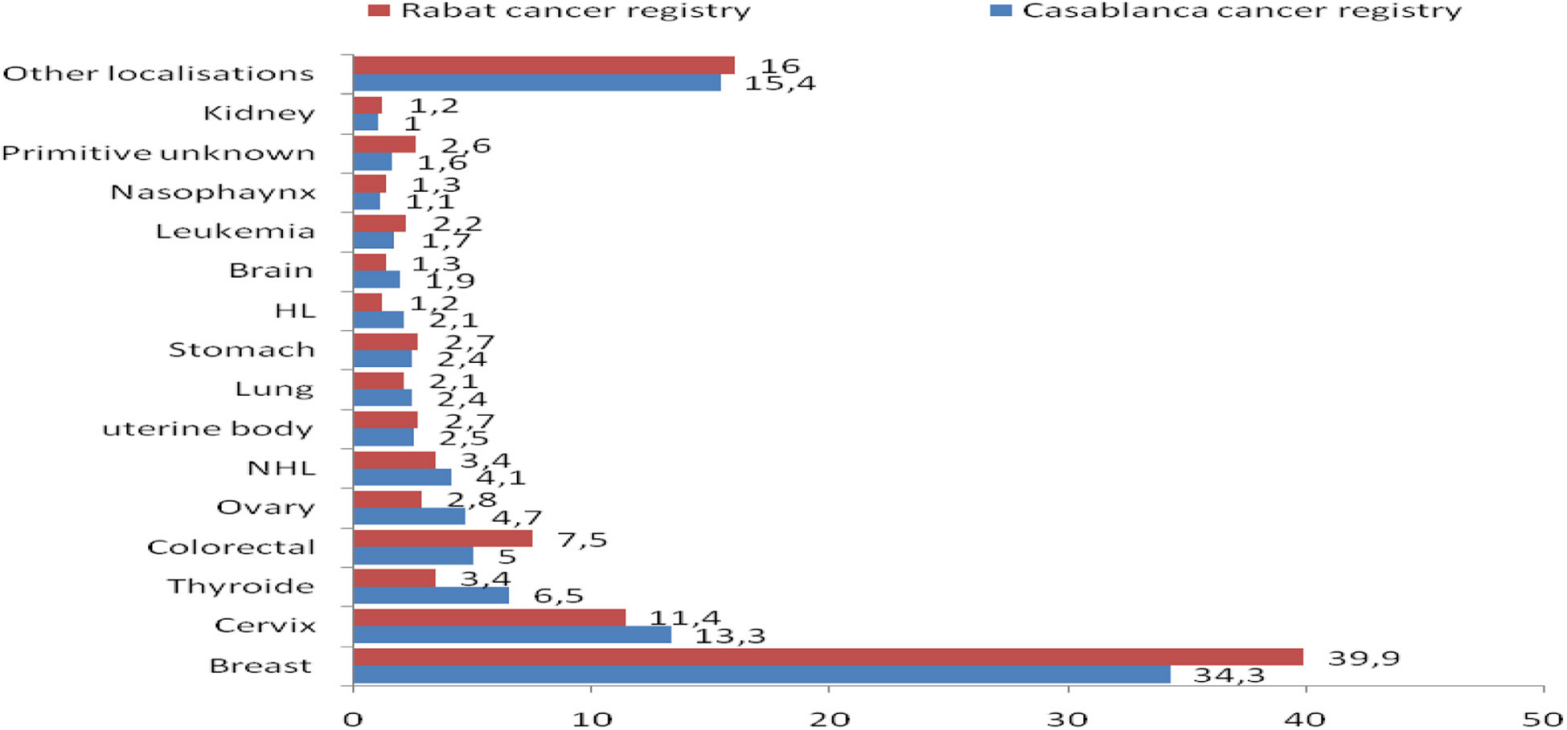
Proportion of cancer cases recorded in women, taken from Casablanca (2005-2007) and Rabat (2006-2008) population-based cancer registries [9, 12]

The four most common cancers sites among males were lung (ASR = 25.9 per 100000), prostate (15.6), bladder (10.2) and colon-rectum (8.4) (proportions of Casablanca cancer registry: 22.1 %, 10.5 %, 7.0 % and 7.2 % of all cancers respectively (Fig. 1, Table 2), whereas in females, the top four cancers sites were breast (ASR = 38.6), cervical (14.6), thyroid (7.1) and colon-rectum (6.0) (proportions of Casablanca cancer registry: 34.3 %, 13.3 %, 6.5 % and 5.0 % of all cancers respectively (Fig. 2, Table 2). The Rabat cancer registry revealed the same pattern with regards to cancer localizations (Figs. 1 and 2).

As shown in Table 3, the mean of age was 49.5 years for breast cancer and 52.9 years for cervical cancer. In men, it was 59.5 years for lung cancer and 70.4 years for prostate cancer. For both sexes, the median age was around 57 years for colon cancer. Breast and cervical cancers are diagnosed at stages II (59.0 %) and III (65.7 %) in women, and only 4.6 % of lung cancers are diagnosed at stages I and II in men (Table 3).

**Table 3:** Age and cancer stage distributions of main cancers in Morocco (2004–2008: data from Casablanca and Rabat cancer registries) [9, 12]

	Age (means, years)		Stage I	Stage II	Stage III	Stage IV	Not defined
	Men	Women					
Breast	-	49.5	13.5	44.6	24.4	4.5	13.0
Cervix	-	52.9	15.0	38.6	27.1	7.9	11.4
Lung	59.5	-	2.3	2.3	19.1	50.0	26.3
Prostate	70.4	-	0.5	19.8	14.1	17.7	47.9
Colon	56.8	57.2	6.2	22.7	14.8	37.5	18.8
Rectum	58.1	54.2	8.2	16.5	12.3	26.0	37.0

According to the Ministry of Health sources, cancer is only second to cardiovascular diseases as the leading cause of death in Morocco since 2004 [16] with a mortality rate of 8.3 %. Cancer remained the second cause of mortality through 2011 as well (11.5 %) [7].

According to GLOBOCAN 2012, 14.1 million new cancer cases and 8.2 million cancer deaths occurred in 2012, compared to 12.7 million and 7.6 million respectively in 2008 [2].

Lung (1.8 million cases, which is 13.0 % of the total), breast (1.7 million cases, or 11.9 % of the total) and the colorectal cancer (1.4 million cases, or 9.7 % of the total) were the most commonly diagnosed cancers. The most frequent causes of death from cancer were lung (1.6 million deaths, 19.4 % of the total), liver (0.8 million deaths, 9.1 % of the total) and stomach (0.7 million deaths, 8.8 % of the total) cancers [2].

### Predictive cancer data of Morocco

The projections based on the GLOBOCAN 2012 estimations show a substantial increase of 19.3 million new incidence of cancer each year by 2025 because of the demographic growth and the ageing of the world population [2]. For Morocco, GLOBOCAN 2012 estimated 35,018 new cancer incidence, (16,829 new incidence in males and 18,189 in females) and 22,798 cancer-related deaths (12,462 in males and 10,336 in females) (Tables 4 and 5). GLOBOCAN 2012 online analysis [2], estimated about 20,715 and 16,942 cancer-related deaths, respectively, in male and female by 2030 (Table 5). This provisional data represents an increases of up of 60 % from 2012 (assuming no change in risk from 2012).

**Table 4:** Data on cancer rates in Morocco estimated by Globocan 2012 [2]

	Men	Women	Both sexes
Population (thousands)	15956	16641	32598
Number of new cancer cases (thousands)	16.8	18.2	35.0
ASR (W)	122.7	114.4	117.8
Risk of getting cancer before age 75 (%)	13.6	11.7	12.6
Number of cancer deaths (thousands)	12.5	10.3	22.8
ASR (W)	92.3	66.8	78.4
Risk of dying from cancer before age 75 (%)	10.2	7.3	8.6
5-year prevalent cases, adult population (thousands)	30.6	49.0	79.6
Proportion (per 100000)	268.4	398.9	336.0

*ASR (W)* Age Standardized Rate (Standardized incidence on World population)

**Table 5:** Number of new cases and cancer related deaths of all cancers excluding non-melanoma skin cancer (all ages) estimated by predictions analysis of Globocan 2012 [2]

Years	Number of new cases			Number of cancer-related deaths		
	Man	Woman	Both sexes	Man	Woman	Both sexes
2012	16829	18189	35018	12462	10336	22798
2025	23665	24802	48467	17441	14631	32072
2030	27354	27894	55248	20715	16942	37657
2035	30893	30765	61658	23944	19264	43208

## Discussion

The surveillance data for cancer were collected from cancer registries. In general, the information was regularly and carefully scrutinized [9, 12]. The incidence rates reported by both population-based cancer registries were considered more accurate than those made by GLOBOCAN. In practice, GLOBOCANestimates sex- and age-specific incidence rates of cancer in Morocco as the weighted average of the local rates of 16 countries [2].

In Morocco, no valid data of mortality due to cancer is currently available. The interpretations and accuracy of cancer mortality patterns presented in this review could be affected by incomplete information on routine medical records and death certificates and inaccurate population estimates. National studies are in progress to collect mortality data in Morocco, but they are not published yet. Only a national study which aimed to provide first data on smoking attributable mortality in Morocco was published in 2014 [17].

Unlike western countries, most Asian and African countries (including Morocco) do not rely on comprehensive death registration systems. Instead mortality data are collected and made available through the GLOBOCAN database by the IARC, which provides data from national incidence estimates using modeled survival for instance, the estimations for Morocco are from national incidence estimates of 82 countries [2]. Major advantages in using this method include national coverage and long-term availability.

The Moroccan population-based cancer registries provide main surveillance data of cancer which have an important roleto ensure reliable and valid information for decision makersin prevention and control strategies. The existing cancer registries in Morocco cover less than 15 % of the Moroccan population, thus still there is a limitation on cancer data obtained from the present registries and the need for more information on cancer in the other regions covering the remaining 85 % of the population is apparent.

Nevertheless, for some countries including Morocco, incomplete coverage of the population yields lowestimated mortality rates whereas for others, the quality of information for the cause of death is poor.

Despite these limitations, data reported from population-based cancer registries and GLOBOCAN database contribute to allow a description of the cancer incidence and trends in Moroccan population.

Lung cancer is the most common cancer affecting males, followed by prostate cancer, while breast cancer represents the most common cancer among females, followed by cervical cancer. Incidence of bladder and colorectalcancer appears to be increasing among Moroccan population.

In general, more than half of the cancers (56.8 %) and deaths due to cancer (64.9 %) in 2012 were registered in the least developed regions in the world, and this trend is likely to continue by 2025 [2]. The median age of all cancer types in Morocco was50.5 years for women and 56.6 years for men. Cancer at a young age in Morocco was more common among women, who represented 67 % of the cases under 50. This was mainly attributable to the low number of cases recorded among old people and the young age of the general population [11, 13].

The pattern of cancer risk as seen in the recent study conducted in North Africa (Morocco, Algeria, Tunisia, Libya and Egypt) appears quite uncharacteristic [18]. In every North Africa country, the whole incidence rate ranges, in both genders, between one third and one half of what is currently measured in Europe. For the whole incidence rate, the variations among these countries are far smaller than the difference between this group of countries and Europe [19].

In Morocco, breast cancer remains the most common malignant tumor in females [9, 12]. Stage at diagnosis of this cancer could be obtained for 82 % cases in 2007. It was recorded in Casablanca cancer registry as follows: 28 % local, 63 % regional and 9 % distant, in the absence of screeningprogram [9, 11].

Breast cancer is also the leading cause of death from cancer in European women. A marked decline in breast cancer mortality in most European countries in the 1990s have been attributed to the combined effect of earlier detection and improved treatment mainly in young women. Soas the population grows older the number of deaths from breast cancer is still rising (131,000 in 2012 [20] and 131,000 in 2013) [21].

Following this European strategy and in order to respond to preventive objectives of National cancer program, Morocco integrated screeningactivities at all levels of its health care system in several regions in 2011 in an effort to reduce breast cancer mortality. These screening programs, focused on the 49–55 years age group,also strive forthe early detection of cervicalcancer [22]. These programsfollow the GLOBOCAN 2012 strategy, which brings to light significant trends of cancer in women and shows that, at the global level, higher priority must be given to prevention andtreatment measureson breast and cervical cancers [2].

WHO has developed guidelines and policies for establishing effective national cancer control programs according to capacity and financial development, in order to increase cancer control activities [23, 24].

Development of a cancer control program should include the implementation of cancer registries to assess the cancer burden, identify priorities and to evaluate the effectiveness of the cancer program.

## Conclusion

The Moroccannational cancer program should aim for coherent and comparableincidence data between differentcancer registries in the country, and develop uniform/homogeneous datasets with respect to quality. Additionally, population-based cancer registries should rapidly recordcancer mortality data including causes of death. It is now important to improve the surveillance of cancer through the implementation of high-quality cancer registries. Thus, the national cancer program should develop cancer registries in more regions to improve our knowledge on cancer epidemiology (particularly in rural areas) and to adapt, if needed, the nationalMoroccan strategy to better control and cure cancer.
